# Triple-blinded randomized clinical trial comparing efficacy and tooth sensitivity of in-office and at-home bleaching techniques

**DOI:** 10.1590/1678-7757-2020-0794

**Published:** 2021-10-01

**Authors:** Sandrina Henn DONASSOLLO, Tiago Aurélio DONASSOLLO, Sumaia COSER, Sabrina WILDE, Juliana Lays Stolfo UEHARA, Luiz Alexandre CHISINI, Marcos Britto CORREA, Maximiliano Sérgio CENCI, Flávio Fernando DEMARCO

**Affiliations:** 1 Universidade Federal de Pelotas Programa de Pós-Graduação em Odontologia Rio Grande do SulPelotas Brasil Universidade Federal de Pelotas, Programa de Pós-Graduação em Odontologia, Rio Grande do Sul, Pelotas, Brasil.; 2 Faculdade Especializada na Área da Saúde do Rio Grande do Sul Faculdade Especializada na Área da Saúde do Rio Grande do Sul,; Faculdade de Odontologia Rio Grande do SulPasso Fundo Brasil Faculdade de Odontologia, Rio Grande do Sul, Passo Fundo, Brasil.; 3 Universidade do Vale de Taquari LajeadoRio Grande do Sul Brasil Universidade do Vale de Taquari – UNIVATES, Lajeado, Rio Grande do Sul, Brasil.; 4 Universidade Federal de Pelotas Rio Grande do SulPelotas Brasil Universidade Federal de Pelotas, Rio Grande do Sul, Passo Fundo, Pelotas, Brasil.

**Keywords:** Clinical trial, Tooth bleaching, Hydrogen peroxide, Carbamide peroxide, Color

## Abstract

**Objective:**

Our study aims to compare the efficacy and tooth sensitivity following in-office (35% hydrogen peroxide) or at-home (10% carbamide peroxide) bleaching treatments both preceded by 2% potassium nitrate (2%KF) desensitizing gel.

**Methodology:**

130 volunteers were randomly allocated to a) in-office bleaching and a placebo at-home protocol; or b) in-office placebo and at-home bleaching treatment. 2% KF was applied for 10 min before both treatments.

**Objective:**

color evaluation was performed (spectrophotometer CIEL*a*b* system and CIEDE2000) to calculate the color change (ΔE00). Subjective evaluation was performed using the VITA classical shade guide followed by shade variation (ΔSGU) at the beginning and end of bleaching treatment and 2 weeks post-bleaching. Tooth sensitivity was daily recorded using a Likert scale varying from 1 (no sensitivity) to 5 (severe sensitivity). Analysis was carried out using non-parametric tests.

**Results:**

Regarding the color change, at-home bleaching resulted in significant color improvement compared to in-office treatment for the parameters Δb* (p=0.003) and Δa* (p=0.014). Two weeks post-bleaching, the at-home treatment resulted in significant color improvement compared to in-office treatment for the parameters Δb* (p=0.037) and ΔE00 (p=0.033). No differences were observed in either ΔSGU parameters. Concerning sensitivity, patients treated with in-office bleaching reported more tooth sensitivity than the at-home group only on the first day after bleaching started, without significant differences in the other periods evaluated (p>0.05).

**Conclusions:**

At-home and in-office bleaching, preceded by a desensitizing agent, were effective for vital teeth bleaching and 10% carbamide peroxide produced a higher whitening effect than 35% hydrogen peroxide in the short time evaluation. Tooth sensitivity rates were similar for the two techniques tested.

## Introduction

Tooth bleaching is the most common esthetic treatment requested by individuals.^1,2^ Many techniques and products are available for tooth bleaching, but at-home bleaching using low concentration gel (10% carbamide peroxide - CP) in a custom tray is still considered the gold-standard treatment for tooth discoloration in vital teeth.^3,4^ This treatment has been effective in producing whiter teeth – lasting up to 2 years without color reversal^6^ – with none or mild transient tooth sensitivity and it is well accepted by patients.^4,5^ Some patients, however, present difficulties in adaptation to at-home protocol, since they prefer not to use a bleaching tray or are not willing to wait 2-3 weeks to see the results, wanting a faster bleaching effect. In these cases, in-office bleaching could be an alternative.^7^

In-office tooth bleaching is performed using high concentration agents (usually 35% hydrogen peroxide – HP), and it is considered safe, efficient and could provide a faster result compared to at-home treatment.^8^ However, higher levels of tooth sensitivity have been related to in-office bleaching.^9,10^ The comparison of at-home and in-office techniques showed similar results, for up to 2 years, concerning bleaching effectiveness, but in-office technique produced higher sensitivity in the initial periods.^11^ Desensitizing agents have been recommended to avoid tooth sensitivity during the bleaching procedures, with potassium nitrate being one of these agents.^12^ A meta-analysis has reported that both potassium nitrate and sodium fluoride were effective in reducing tooth sensitivity,^13^ despite the contradictory results observed in recent studies.^12,14,15^

Randomized clinical trials have compared the in-office and at-home dental bleaching, mainly with single and double-blind designs.^6,11,16-18^ Studies with triple-blind randomized design have, in general, only evaluated one technique (either at-home or in-office)^19,20^ due to the participants’ blinding difficulties. Therefore, there is no known triple-blind randomized clinical trial comparing different techniques of bleaching (at-home or in-office tooth bleaching) preceded by the use of potassium nitrate.

Our study aims was to compare the efficacy (color change) and adverse effect (tooth sensitivity) produced by in-office and at-home bleaching treatments preceded by 2% potassium nitrate (2%KF) desensitizing gel. The hypothesis is that both bleaching treatments would produce similar results concerning efficacy, and in-office bleaching would cause higher sensitivity.

## Methodology

### Trial design

This study was a randomized, triple-blind, clinical trial with an equal allocation rate to receive either one of two treatments, following the guidelines published by Consolidated Standards of Reporting Trials-CONSORT,^21^ and approved from the Ethics Research Committee of the University of Cruz Alta, under number 462.122. The study was conducted for Faculdade Especializada na Área da Saúde do Rio Grande do Sul, a medium-sized city on Passo Fundo, and all subjects signed an informed consent form.

### Training of examiner

Objective and subjective methods were used to evaluate tooth color. One examiner was trained^22^ on shade determination of anterior teeth in 10 subjects using a digital spectrophotometer (Vita Easyshade, Germany) and Vita shade guide units (SGU) (VITA classical A1-D4^®^ shade guide, Vita Zahnfabrik). Subjective evaluation was initially performed followed by the objective evaluation, which was made in the middle third of the upper two central incisors, three times. For this, a custom tray was made with an orifice to standardize the location of the color measuring.

### Tooth shade measurement

The primary outcome of this study is color change. Thus, the tooth color coordinates, based on the CIEL* a* b* system, were objectively measured using a pre-calibrated digital spectrophotometer (Vita Easyshade; Vita Zahnfabrik). At each evaluation period, the shade of the upper two central incisors was measured three times, with the active point of the instrument at the place determined by custom tray (middle third).^22,23^ The spectrophotometer automatically averaged the parameters evaluated, L*, a* and b*. The L* represents the lightness. The a* value is a measure of redness (positive a*) or greenness (negative a*). The b* value is a measure of yellowness (positive b*) or blueness (negative b*). The average value was estimated and recorded. The color difference (ΔE^00^) between any 2 measurements was estimated using the CIEDE2000 metric: $\Delta E^{00}=[{\left(\Delta l^{\prime }/K_{L}S_{L}\right)}^{2}+(\Delta C^{\prime }/{{K_{c}S_{c})}^{2}+{\left(\Delta H^{\prime }/K_{H}S_{H}\right)}^{2}+R_{T}\left(\Delta C^{\prime }/K_{c}S_{C}\right){\left(\Delta H^{\prime }/K_{H}S_{H}\right)}^{2}]}^{1/2}$, in which ∆L′, ∆C′, and ∆H′ are the differences in lightness, chroma, and hue for a pair of samples. R_T_ is the rotation function that accounts for the interaction between chroma and hue differences in the blue region. S_L_, S_C_, and S_H_ are weighting functions used to adjust the l ΔE^00^ for variation in perceived magnitude, with variation in the location of the color coordinate and differences between the two color readings; and k_L_, k_C_, and k_H_ are the correction terms for the experimental conditions. ΔE^00^ ≥ 1.8 is the acceptable color difference threshold for the CIEDE2000 method.^24^ The subjective evaluation was performed using the VITA classical shade guide (VITA classical A1-D4^®^ shade guide, Vita Zahnfabrik), under the same conditions used for the objective evaluation. A single examiner performed all shade evaluations. The shade difference was estimated for the same tooth before (S1) and after (S2) the bleaching protocols ( ΔS=S2−S1 ). The 16 shade tabs were, in order of lightness, numbered from 1 (highest value, B1) to 16 (lowest value, C4), as follow: B1=1, A1=2, B2=3, D2=4, A2=5, C1=6, C2=7, D4=8, A3=9, D3=10, B3=11, A3.5=12, B4=13, C3=14, A4=15, and C4=16.^22,25^

The differences between the groups were analyzed using the differences in color (ΔE^00^),^25^ lightness (ΔL*), chroma (Δa*), and value (Δb*) as well as those in the Vita shade guide units (ΔSGU) considering two periods: a) final – baseline and b) 2 weeks post-treatment – baseline.

### Sample size

Sample size was estimated based on a previous study^11^ that showed that one week after treatment, the change in tooth color shade from baseline was on average 6.27 with a standard deviation (SD) of 1.5 for at-home bleaching, whereas this change was 5.62 with an SD of 0.9 for the in-office bleaching technique. Considering 80% power and 5% significance level, 108 patients would be required. Another 20% were added to the sample to account for possible losses and refusals, obtaining a total of 130 patients. The individuals were invited to participate in our study by banners fixed both in local colleges and in college’s websites.

### Eligibility criteria

The subjects included in this clinical trial were at least 18 years old and have good oral and general health. Furthermore, volunteers needed to have central incisors without restorations on the labial surfaces and to be shade B2 or darker, according to a value-oriented shade guide, and they should have never undergone tooth bleaching. Participants were excluded from this study if they were smokers, were using braces, had undergone tooth-whitening procedures, were pregnant or lactating, had labial surface restoration on the central incisors, had severe internal tooth discoloration (such as pulpless teeth, fluorosis, and tetracycline stains), presented tooth sensitivity and abrasion, erosion and/or abfraction and recession. Participants’ age varied from 18 to 40 years old, with a mean age of 23.2 (±5.8).

### Randomization and blinding

**Figure 1:**
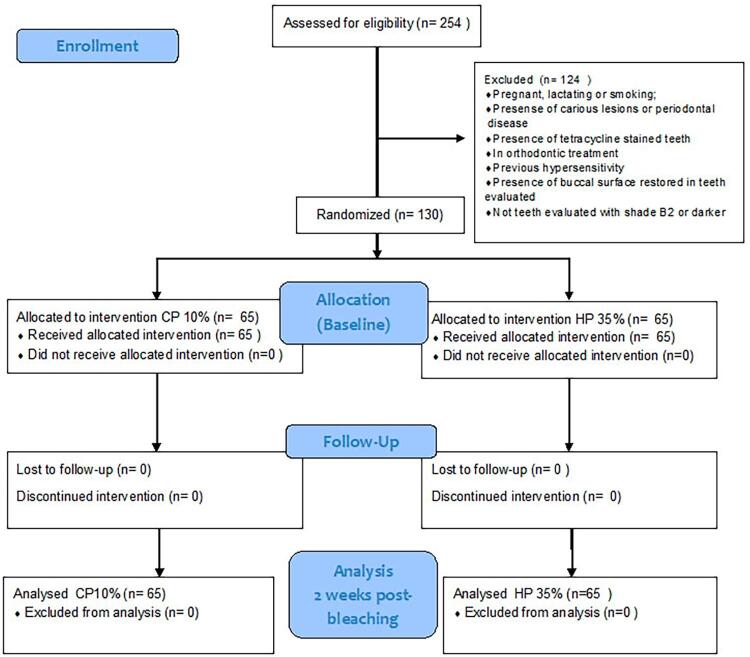
Flow-chart of the trial

The 130 participants were randomly allocated into two groups (n=65) according to the bleaching techniques (in-office and at-home) (Figure 1). For this purpose, the two groups were identified with two different colors: in-office with yellow and at-home with green. A person not involved in the research protocol performed the randomized process using 130 brown envelopes, 65 of which contained yellow paper and the other 65, green paper. The participants took one envelope, and the person not involved revealed the allocation for the other person. Neither the participant nor the operator and examiner knew the meaning of the colors, being blinded to the protocol.

### Groups and placebo

The yellow group received the in-office treatment with 35% hydrogen peroxide gel (Whiteness HP Blue, FGM Dental Products, Brazil) and underwent a placebo at-home treatment using custom-made trays, but using a product similar to at-home gel, formulated without any bleaching agent.

The green group received a placebo in-office treatment with a similar product to active in-office treatment, but without bleaching agent. This group underwent the at-home treatment with 10% carbamide peroxide (Whiteness Perfect, FGM Dental Products, Brazil).

To produce the placebos, the manufacturer (FGM Dental Products, Brazil) provided the tubes without a bleaching gel agent. The placebo was a water-based gel produced in a Drugstore, with the same color and viscosity as the original gel. The placebo gel was inserted into the tubes. A person not involved in the study carried out the manipulation and identification.

The chemical characteristics and manufacturers of tested agents are shown in Table 1.

**Table 1:** Bleaching agents tested, their chemical characteristics and manufacturers

Group	Bleaching agent	Chemical characteristics	Manufacturers	Application technique

At-home	White Perfect	10% Carbamide Peroxide Potassium nitrate Sodium fluoride	FGM	2 hours per day for 14 days
In-office	White HP Blue	35% Hydrogen Peroxide Thickeners Violet Pigment Neutralizing agents Calcium Gluconate Glycol Deionized Water	FGM	one application of 40min for week 2 weeks
Placebo At-home		Carbopol^®^ 940 (10g) Distilled water (11g) AMP 95 (1g)	Drugstore	2 hours per day for 14 days
Placebo In-office		Carbopol^®^ 940 (10g) Distilled water (11g) AMP 95 (1g) Violet Pigment (0,5g)	Drugstore	one application of 40min for week 2 weeks

Potassium nitrate desensitizing gel (Desensibilize KF2%, FGM, Brazil) was applied for 10 minutes on the labial surface for both groups in the first section

### Bleaching procedure

For all participants, an alginate impression of each subject’s maxillary arch was prepared and filled with dental stone. The custom tray was produced using a 1-mm soft vinyl material. The excess of labial and lingual surfaces was cut 1 mm from the gingival junction. After that, all volunteers received prophylaxis in all teeth.

After the color evaluation, the lips, cheeks, and tongue were isolated using a lip retractor (Arcflex, FGM, Brazil). The gingival tissue was isolated using a light-cured resin dam (Top Dam, FGM, Brazil), and the potassium nitrate desensitizing gel (Desensibilize KF2%, FGM, Brazil) was applied for 10 minutes on the labial surface for both groups in the first clinical session. The subject from the yellow group received in-office bleaching with 35% hydrogen peroxide. The bleaching gel was applied to the labial surfaces of teeth for 40 minutes. The bleaching agent was moved every 5 minutes. The in-office bleaching treatment was applied twice, with an interval of seven days. In the first session, the participants received the instructions and started the placebo at-home bleaching (2 hours/day for 2 weeks).

Subjects from the green group received the potassium nitrate desensitizing gel, a placebo in-office bleaching (one application of 40min a week, for 2 weeks). In the at-home bleaching treatment, a custom tray was used, containing 10% carbamide peroxide gel (Whiteness Perfect, FGM, Brazil). All subjects were instructed to wear the tray with the bleaching agent for at least 2 hours/day. After that, subjects were instructed to remove the tray, wash it and brush their teeth with toothpaste. The treatment was conducted for 2 weeks. The color evaluation was carried out at the beginning of treatment (baseline), in the end of bleaching treatment (final), and 2 weeks post-bleaching.

### Tooth sensitivity data

Tooth sensitivity is the secondary outcome of the study. Besides the bleaching, participants were asked to record, daily, their tooth sensitivity, according to a 5-point Likert scale with the following criteria: 1= none, 2= mild, 3= moderate, 4= considerable, and 5= severe.^26^

### Statistical analysis

Statistical Analysis was performed using Stata 14.0 (StataCorp, College Station, Texas, US.). Prior to tests, data were checked for normality. The normal distribution of data was not observed and a non-parametrical analysis was performed. Friedman test followed by post hoc Tukey test was used to analyze differences within treatment groups between different points of follow-up. Differences between groups were assessed using Mann Whitney test. Differences were considered statistically significant when p<0.05.

## Results

One hundred and thirty subjects have completed the study, with 65 volunteers allocated to each group. There were no dropouts during the evaluation process. Seventy-four (56.9%) patients were females. At baseline, treatment groups presented a similar proportion according to age, gender, profession, and education level (Table 2).

**Table 2:** Demographic characteristics according to the different treatment groups

Variables	Categories	In-office	At-home

Gender	Male	32 (49%)	24 (37%)
	Female	33 (51%)	41 (63%)

Age (years)	≤ 20	27 (41.6%)	22 (33.8%)
	21-22	7 (10.8%)	12 (18.4%)
	23-24	12 (18.4%)	9 (13.9%)
	25-26	6 (9.2%)	7 (10.8%)
	≥27	13 (20%)	15 (23.1%)

Education level	Middle and high school	23 (35.4%)	22 (33.8%)
	Complete college	10 (15.4%)	16 (24.6%)
	Incomplete college	32 (49.2%)	27 (41.6%)

Profession	Student	35 (53.8%)	34 (52.3%)
	Liberal professions	23 (35.5%)	24 (36.9%)
	Public server	7 (10.7%)	7 (10.8%)

### Color change

Results of the study for L*(lightness), a* (redness), and b*(yellowness), for group treated with 10% carbamide peroxide (at-home) and 35% hydrogen peroxide (in-office) are shown in supplementary table S1 and S2, respectively. The median values (SD) for ΔL*, Δa*, Δb*, ΔE^00^and ΔSGU from the at-home and in-office groups are shown in Table 3.

**Table 3:** Comparison of in-office and at-home bleaching by different color coordinates of CIEL*a*b* system, ΔE00 and ΔSGU

Tooth color parameters	Evaluation period	In-office bleaching	At-home bleaching	P value

ΔL	Final	1.73 (3.29)	0.79 (3.99)	0,159
	2 weeks-post bleaching	-0.14 (6.50)	0.91 (5.36)	0,173
Δa	Final	0.33 (1.54)	-0.09 (1.42)	0.014*
	2 weeks-post bleaching	-0.13 (1.67)	-0.13 (1.25)	0,176
Δb	Final	-2.16 (2.68)	-3.55 (5.89)	0.003*
	2 weeks-post bleaching	-2.99 (2.59)	-4.25 (4.19)	0.037*
ΔE00	Final	4.03 (1.77)	4.33 (2.35)	0,083
	2 weeks-post bleaching	4.01 (1.02)	4.27 (1.59)	**0,033**
ΔSGU	Final	-3.00 (2.18)	-3.5 (2.27)	0,669
	2 week-post bleaching	-3.00 (2.17)	-4.00 (2.29)	0,445

* Differences were considered statistically significant when p<0.05. Mann Whitney test was applied for statistical comparison.

### Final stage of treatment

No significant difference was observed for ΔL* (=0.159), ΔE^00^ (p=0.083), and ΔSGU (p=0.669) between groups at the end of treatment. At-home bleaching resulted in significant color improvement compared to in-office treatment for the parameters Δb* (p=0.003) and Δa* (p=0.014).

### 2 weeks post-bleaching

No significant difference between in-office and at-home bleaching was observed for ΔL* (p=0.173), Δa* (0.176) and ΔSGU (p=0.445) parameters, 2 weeks post-bleaching period. At-home bleaching resulted in significant color improvement compared to in-office treatment for the parameters Δb* (p=0.037) and ΔE^00^ (p=0.033).

### Tooth sensitivity

Table 4 shows the comparison between tooth sensitivity for the two groups. More sensitivity was reported by the in-office group, when compared with at-home bleaching, only on the first day of evaluation (p<0.05). In the other periods evaluated, no significant differences were observed between groups (p>0.05). A significant decrease in sensitivity was detected in both groups, after the bleaching completion.

**Table 4:** Means (SD) values for weekly tooth sensitivity and degrees of tooth sensitivity reported by volunteers in different treatment groups

Treatment	First day	1 week	2 weeks	1 week post-bleaching	2 weeks post-bleaching

In-office	1.57 (0.84)^Aa^	1.24 (0.36)^Aa^	1.31 (0.43)^Aa^	1.07 (0.27)^Ab^	1.00 (0.00)^Ab^
At-home	1.20 (0.51)^Ba^	1.37 (0.53)^Ab^	1.31 (0.54)^Aab^	1.02 (0.13)^Aac^	1.02 (0.13)^Aac^

* Different uppercase letters indicate differences between groups in each time of evaluation. **Different lowercase letters indicate differences between different periods of evaluation within each treatment group.

Table 5 shows the description of the type of sensitivity observed for both groups. Most of the sensitivity observed was classified as mild and occurred during bleaching treatment (1 and 2 weeks). One and 2 weeks post-bleaching, the participants reported practically no sensitivity. During bleaching procedures, few subjects reported moderate and sporadic cases of severe discomfort, but no individual requested the desensitizing agent to use during the treatment.

**Table 5:** Degrees of tooth sensitivity reported by volunteers in different treatment groups

Degree		First day	1 week	2 weeks	1 week post-bleaching	2 weeks post-bleaching

At-home	None	55 (84.6%)	31 (47.7%)	38 (58.5%)	62 (95.4%)	63 (96.9%)
	Mild	7 (10.8%)	23 (35.4%)	19 (29.2%)	3 (4.6%)	2 (3.1%)
	Moderate	3 (4.6%)	5 (7.7%)	7 (10.8%)	-	-
	Considerable	-	5 (7.7%)	-	-	-
	Severe	-	1 (1.5%)	1 (1.5%)	-	-

In-office	None	41 (63.1%)	34 (52.3%)	34 (51.4%)	60 (92.3%)	65 (100%)
	Mild	13 (20%)	21 (32.4%)	21 (31.4%)	3 (4.6%)	-
	Moderate	10 (15.4%)	09 (13.8%)	6 (10%)	2 (3.1%)	-
	Considerable	-	-	4 (7.2%)	-	-
	Severe	1 (1.5%)	1 (1.5%)	-	-	-

## Discussion

The hypothesis tested in the study was rejected, since at-home bleaching showed a slightly better result than in-office bleaching protocol. Both treatments were effective to make teeth whiter, but more color improvement was observed for 10% carbamide peroxide in most of the parameters evaluated in the short period of follow-up. Even though these results were statistically significant, considering the small difference between the 2-weeks post bleaching techniques, the better performance observed for the at-home bleaching could not be clinically observed. Thus, these results should be interpreted carefully. More sensitivity was reported for in-office treatment, but only on the first day of bleaching therapy. Another study comparing these two bleaching protocols found that both were effective in whitening the teeth, but the authors observed that in-office bleaching was associated with higher tooth sensitivity.^11^ Also comparing in-office (35 and 38% HP) with at-home (10 and 20% CP), Basting, et al.^10^ (2012) observed that all protocols were effective to bleach teeth, without differences regarding final color shade. A recent systematic review and meta-analysis comparing at-home and in-office techniques – including 12 studies for qualitative evaluation and 8 studies for quantitative analysis – was not able to show any significant difference between the two techniques regarding the effectiveness in improving the color and the sensitivity produced. The authors attributed the lack of differences due to the high variability in the protocols used to carry out bleaching in both techniques.^27^

The efficacy of bleaching agents relies on the release of free oxygen, which could break down the pigments present in the tooth structure producing a whitening effect.^6^ To explain the better result observed for at-home bleaching, we could hypothesize that, despite the lower concentration of bleaching agent compared to 35% hydrogen peroxide, using 10% carbamide peroxide in the custom tray allows the product to be in constant contact with the tooth surface. Some studies have reported that a more concentrated agent used for in-office bleaching would produce a faster bleaching effect, which was not observed in our study. In the present study, we used in-office bleaching without a light source to increase the whitening effect. The results from different systematic reviews and meta-analyses have not shown any significant effect when using a light unit to improve the bleaching effect for a high concentration of hydrogen peroxide.^28-30^ Also, a recent network meta-analysis included 18 studies in the quantitative synthesis and observed no superior effect of in-office bleaching with the use of any light activation.^31^ Our study contemplates only the first two weeks after bleaching treatment; the patients are to be followed aiming to observe possible differences between treatments in a long term. A longer follow-up is needed to determine the durability of the treatment and the potential for color reversal.^6^

Color evaluation in this study was performed with subjective and objective methods. The subjective assessment is important for color research and can be characterized by perceptibility and acceptability thresholds. While the subjective visual scale is the system most commonly employed by clinicians to reproduce specific shades, this technique still presents a challenge for clinical Dentistry^23^ and can be influenced by evaluators’ characteristics (gender, eye fatigue, and experience).^32^ To avoid possible imprecisions, we also perform the analysis with a digital spectrophotometer using the CIEDE2000 system.^33-36^ The spectrophotometer data from both groups were able to show differences in almost all parameters after bleaching. Parameter of ΔE^00^, 2 weeks post-bleaching, presents slightly color improvement in at-home bleaching. Clinically relevant bleaching effect can improve the Oral Health-Related Quality of Life in individuals with dark teeth that underwent bleaching treatments.^16^ A multicentric study observed that 50% of perceptibility and acceptability thresholds of color change in CIEDE2000 was 0.81 (95% CI 0.34 – 1.28) and 1.77 (95% CI 1.23 – 2.37), respectively.^36^ In our study, both bleaching treatments presented color change values in CIEDE2000 parameters higher than the aforementioned values. However, the differences between the treatments have remained below these limits, meaning that both treatments showed perceived changes but perhaps the change between treatments was so subtle that it could not be clinically perceived.

The most common adverse effect in vital bleaching reported by patients is tooth sensitivity.^13^ This sensitivity has been related to the increased porosity produced by bleaching agents, which allow the penetration of ions and liquid changes into the dentinal tubules that could cause sensitivity.^37^ Additionally, it was hypothesized recently that a chemo-sensitive ion channel-TRPA1 could be sensible to a variety of oxidizer compounds including hydrogen peroxide. The activation of intradental nerve activity via TRPA1 could be the mechanism of pain decurrent of bleaching treatment.^38^ Even though there is a high prevalence of sensitivity after bleaching treatment, the degree of sensitivity has mostly been reported to be mild.^39^ Indeed, in our study, the prevalence of sensitivity in both groups had the peak around the first week during bleaching treatment, when almost 50% in each group had experienced pain, mostly of mild and transitory intensity, as previously reported.^11^ Some clinical trials have found no difference in tooth sensitivity with the use of potassium nitrate as a desensitizing agent.^15,19^ Although, Martini, et al.^15^ (2020) observed that 2% potassium nitrate was able to reduce the sensitivity when applied before, or both before and after the bleaching, the topical application of 10% potassium nitrate before in-office bleaching did not reduce neither the risk nor the intensity of tooth sensitivity in a randomized clinical trial.^12^ Other randomized clinical trials have observed that desensitizing agents could reduce sensibility,^40,41^ since a meta-analysis detected significant results in favor of the use of potassium nitrate.^13^ In our study, the direct effect of potassium nitrate was not evaluated. Differences in tooth sensitivity are possibly linked to the bleaching technique itself, since both bleaching techniques were preceded by desensitizing agent application.

In a double-blind randomized clinical trial, subjects treated with in-office bleaching reported a higher intensity of tooth sensitivity than those individuals who underwent at-home bleaching treatment.^11^ In our study, the only noticeable difference between the two protocols was in the first day of treatment, when patients from in-office bleaching reported a significantly higher mean of pain. Such finding agrees with previous studies, in which tooth sensitivity due to bleaching usually occurred within the first 24 hours.^39,42,43^ We argue that the higher concentration level of peroxide causes more porosity – at least in the first few hours – consequently provoking some discomfort. However, no significant difference was observed between treatments after these initial results. The discomfort was reduced over time and sensitivity almost disappeared with the cessation of bleaching treatments. We emphasize that, in our study, patients that reported a more severe degree of discomfort during treatment did not request treatment for their discomfort, nor did they request interruption of treatment.

Randomized clinical trials are the best study design to show the efficacy of treatments, especially when they follow specific guidelines.^44^ We have followed the guidelines for an RCT and the study was reported using the Consort recommendations. Additionally, the study has an adequate sample size, the randomization was carried out to guarantee the similarity between groups before treatment starting and the blinding process was able to avoid that volunteers, operators, and evaluators could be informed about the treatments, which is important to prevent bias. Some limitations, however, need to be cited. First, tooth sensitivity was a secondary outcome and there was no group without the application of the desensitizing agent before the bleaching treatments. Therefore, the direct influence of the desensitizing agent on tooth sensitivity was not evaluated. Second, the subjects were from a private university, with a higher socioeconomic status compared to the general population, which limits the external validity of our study. However, tooth bleaching is one of the most required procedures in private dental offices, which are usually attended by individuals with higher socioeconomic levels. Therefore, our results could be in line with the potential results observed in these private practices. We also observed small differences concerning color parameters between the two techniques. We could not, however, ensure that patients with at-home bleaching have higher levels of satisfaction, since our study did not evaluate this parameter. Moreover, our results cannot be extrapolated over long periods, since the participants were only followed for two weeks post-bleaching.

Our randomized clinical trial was able to show that both bleaching treatments, preceded by the use of 2% potassium nitrate, had efficacy to bleach teeth with minimum adverse effects. At-home bleaching protocol produced slightly better results than in-office treatment according to Δa*and Δb* parameters. At-home bleaching uses less aggressive agents and usually presents a lower cost than in-office treatment^45,46,47^. When used as recommended by the professional^45,47^, this treatment seems to be the first-choice therapy to treat discolored vital teeth. Indeed, when evaluating the preferences of Brazilian dentists for vital tooth bleaching,^45^ at-home bleaching was preferred over in-office therapies and 10% CP was the most selected agent.

## Conclusions

The results of our study suggested that both techniques – at-home and in-office bleaching, following the use of 2% potassium nitrate – were effective for vital teeth bleaching. However, the 10% carbamide peroxide produced a better whitening effect than the 35% hydrogen peroxide in the short term evaluation. The tooth sensitivity rates were low and similar for the two techniques tested.

## Supplementary tables


