# Supplementary material for: Triple-blinded randomized clinical trial comparing efficacy and tooth sensitivity of in-office and at-home bleaching techniques
Source: J Appl Oral Sci. 2021 Oct 1;29:e20200794. doi: 10.1590/1678-7757-2020-0794 (PMC8523096; doi:10.1590/1678-7757-2020-0794)
Supplement: Supplementary file 1 [file 1678-7757-jaos-29-e20200794-supp01.pdf]

Supplementary tables

Table S1- Comparison of different color coordinates of CIEL*a*b* system for at-home bleaching patients group at different times

	Median (25;75 percentiles)		
**Tooth color parameters**	**Baseline**	**Final**	**2 weeks post-bleaching**
L	84.15 (80.45;86.35)^{A}	83.45 (81.43;87.10)^{B}	85.20 (82.38;87.45)^{B}
a	-1.70 (-2.20;-1.20)^{A}	-1.85 (-2.13;-1.45)^{A}	-1.90 (-2.20;-1.45)^{A}
b	14.38 (13.03;16.45)^{A}	10.10 (8.48; 13.20)^{B}	10.20 (8.50;12.80)^{B}

* Differences were considered statistically significant when p<0.05. Different letters indicate differences between group

Table S2- Comparison of different color coordinates of CIEL*a*b* system for in-office bleaching patients group at different times

	Median (25;75 percentiles)		
**Tooth color parameters**	**Baseline**	**Final**	**2 weeks post-bleaching**
L	83.75 (82.15;85.75)^{A}	86.25 (83.65;88.00) B	84.70 (82.40;87.50) A
a	-1.78 (-2.15;-1.25)^{A}	-1.65 (-2.05;-0.80) A	-1.68 (-2.20; -1.25) A
b	14.60 (11.55;16.55)^{A}	12.43 (9.50;14.80) B	11.45 (9.65;13.55) B

* Differences were considered statistically significant when p<0.05. Different letters indicate differences between group
